# Role of Cultured Skin Fibroblasts in Aesthetic and Plastic Surgery

**Published:** 2013-01

**Authors:** Davood Mehrabani, Navid Manafi

**Affiliations:** 1 Stem Cell Research and Transgenic Technology Research Center, Shiraz University of Medical Sciences, Shiraz, Iran; 2 Student Research Committee, Tehran University of Medical Sciences, Tehran, Iran

**Keywords:** Skin, Fibroblast, Aesthetic Surgery

The skin is the largest tissue of man with several functions. Cosmetics/cosmeceuticals agents always are needed to be evaluated for their detrimental effects on the skin. Nowadays, the therapeutic potential of stem cells has and fibroblasts have increased the hope for a successful cell therapy in aesthetic medicine. Stem cells are unspecialized cells capable of renewing themselves through cell division without limit as long as the person is still alive. Each new cell has the potential either to remain a stem cell or become another type of cell.^1^

In a healthy individual, skin integrity is supported by epidermal stem cells that self-renew and generate daughter cells undergoing terminal differentiation. In addition to accumulation of senescence markers in aged skin, epidermal stem cells are maintained at normal levels throughout life. So, skin ageing is provided by an impair in stem cell mobilization or a reduction in the number of stem cells being capable to respond to proliferative signals. The self-renewal and multi-lineage differentiation of skin stem cells make these cells attractive for ageing process studies but also for regenerative medicine, tissue repair, gene therapy and cell-based therapy with autologous adult stem cells.^2^

Healing in skin wound demonstrates an extraordinary mechanism of cascading cellular functions. Migration of fibroblasts along with the fibrin network and start of reepithelialization from the wound edges, angiogenesis and neovascularization get activated by capillary sprouting.^3^ Sakrak *et al.* (2012) noted that cultured fibroblasts, particularly with a dermal support, contribute to the wound healing process; reduce the contraction of the wound; and support collagen synthesis and neovascularization.^4^ Many potential mechanisms exist to impair healing. One contributory mechanism may be inhibition of fibroblast proliferation and induction of a stress-induced premature senescence phenotype by the continuing inflammation found in chronic wounds. They showed that recognition of the role of fibroblast senescence in wound chronicity may allow for identification of those wounds that will respond positively to these products.^5^

Adipose-derived stem cells (ADSC) were shown to have relative advantages in accessibility and abundance compared to other kinds of stem cells in treatment of many dermatologic diseases. Subcutaneous injection of ADSC could significantly increase the collagen synthesis in hairless mice, and dermal thickness, collagen density and fibroblast number, angiogenesis, procollagen type I protein and mRNA expression also increased. They recommended that ADSC therapy may be useful in ageing skin as ADSC have antioxidant, whitening and wound-healing effects in the skin by secretion of growth factors and by activating fibroblasts.^6^ The ADSC and its secretory factors were demonstrated to be effective for UVB-induced wrinkles too, and the antiwrinkle effect was mainly mediated by reducing UVB-induced apoptosis and stimulating collagen synthesis of HDF.^7^ Cell therapy for facial anti-aging in clinical experience was introduced including cultured gingival fibroblasts injection lasting for at least one year, making it a good option for patients.^8^ Park et al. (2008) demonstrated that ADSCs and their secretory factors showed promise for application in cosmetic dermatology, especially in the treatment of skin aging too.^9^

In aging, loss of collagen and elastin are the important visible processes. Schmidt (2011) reported that the US Food and Drug Administration (FDA) approved laViv (azficel-T), a first-in-class personalized cell therapy to remove fine wrinkles or nasolabial folds around the nose and mouth.^10^ Autologous fibroblasts grown in culture (azficel-T) were shown to correct the appearance of aging and wrinkles by replacing lost dermal constituents showing that autologous cell therapy can mark the beginning of a new phase in aesthetic therapy.^11^ Eca *et al.* (2012) showed that injection of skin fibroblasts cultivated in medium supplemented with human serum was a viable method and had no side effects. Four injections at 15-day intervals containing 6.4×10^6^ fibroblasts/mL could significantly improve periorbital skin flaccidity.^12^ In a study by Khodadadi *et al.* (2010) on 10 patients with stable vitiligo, it was shown that intra-epidermal injection of dissociated autologous epidermal cells could improve the patches in 80% of subjects.^13^

Dermal fibroblasts are responsible for synthesizing and organizing the dermis with three layers of (i) epidermis containing keratinocytes, melanocytes, and Langherans cells; (ii) dermis that is populated with fibroblasts, vessels and dendritic cells; and (iii) subcutaneous tissue. A basement membrane separates epidermis from dermis by composed of collagens and laminins, synthesized by fibroblasts and keratinocytes. Type I collagen is the most protein in the dermis produced by fibroblasts, synthesizing other collagens (III, V, VII), elastin, proteoglycans, and fibronectin too. The half-life of type I collagen in human skin was shown to be greater than 1 year.^14^ Fibroblasts have a crucial role in wound healing. They produce matrix metalloproteinases and plasmin too. Its synthesis is increased in remodeling of an injured area after a wound^15^^,^^16^ and also its production has an increasing trend in fibrotic diseases^17^^,^^18^ while has a decreasing trend during aging and after a sun exposure.^19^

For decades, skin organ culture has successfully been used to study skin ex vivo .^20^ Normal human dermal fibroblast cultures can be divided into three phases of (i) primary cultures established by enzymatic digestion of the dermis, or by outgrowth of fibroblasts from explanted tissue pieces; (ii) secondary cultures as actively proliferating cells, provided from passage and expansion of primary cultures and (iii) terminal cultures eventually reaching a state of replicative senescence to be aging at the cellular level.^21^^.^^22^ Cryogenic preservation of low passage-number fibroblasts is useful to maintain reserves of cells for further therapeutic measures. Therefore, it is easy to rapidly build a bank of fibroblasts from a limited number of skin samples. Culture of skin fibroblasts in collagen gels was first described by Bell *et al.*^23^ The collagen gel serves as a stimulus to modulate fibroblast behavior.^24^ In monolayer, fibroblasts are flat, spindle-shaped, and organized in parallel arrays, but in a three-dimensional collagen gel, they are elongated and have several dendrites. Fibroblasts in collagen gels usually proliferate slower than in monolayer cultures.^25^ They are cultured in Dulbecco’s MEM supplemented with non-essential amino acids and 10% fetal bovine serum (DMEM-FBS). Cell growth is undertaken at 37◦C in 5% CO2 and 95% air. Fibroblasts are subcultured using trypsin/EDTA as required, and are used at passage 3 to 5.^26^ Therefore, it seems that autologous fibroblasts are good sources to correct the appearance of aging and wrinkles by replacing lost dermal constituents and have a crucial role in healing showing that the autologous cell therapy can be a new phase in aesthetic therapy with no side effects for the patient.

## CONFLICT OF INTEREST

The authors declare no conflict of interest.

## Footenote

Please cite this paper as:

Mehrabani D, Manafi N. Role of Cultured Skin Fibroblasts in Aesthetic and Plastic Surgery. World J Plast Surg 2013;2(1): 2-5.
